# Effect of consuming food cooked in iron pots and behavior change communication on iron status in non-pregnant, non-lactating women: protocol for randomized controlled trial

**DOI:** 10.1186/s13063-025-09202-0

**Published:** 2025-10-31

**Authors:** Umesh Kawalkar, Shital Telrandhe, Mahalaqua Nazli Khatib, Shilpa Gaidhane, Zahiruddin Quazi Syed, Amar Mankar, Abhay Gaidhane

**Affiliations:** 1 Department of Community Medicine, Government Medical College and Hospital, Akola, Maharashtra, India; 2 Datta Meghe Institute of Higher Education and Research, Wardha, Maharashtra India

**Keywords:** Anemia, Iron-deficiency, Randomized controlled trials as topic, Cooking, Iron, Dietary, Hemoglobin, Ferritin, Patient compliance, Rural population

## Abstract

**Background:**

Iron-deficiency anemia (IDA) remains a significant public health concern, particularly among women of reproductive age in developing countries. Despite ongoing supplementation and fortification programs, the prevalence of anemia remains high due to challenges such as poor compliance, logistical constraints, and limited access to iron and dietary sources. Cooking in iron cookware has been proposed as a low-cost, culturally acceptable strategy for improving dietary iron intake; however, evidence of its effectiveness remains mixed. This randomized controlled trial (RCT) aimed to evaluate the effectiveness of consuming food cooked in iron pots, coupled with a structured behaviour change programme, on improving hemoglobin and ferritin levels among non-pregnant, non-lactating women of reproductive age in rural India.

**Methods:**

A community-based randomized controlled trial will be conducted in selected rural blocks of Akola and Wardha districts in Maharashtra, India. Eligible women (aged 18–45 years, non-pregnant, non-lactating) will be randomized into two groups: an intervention group receiving iron cookware along with structured behaviour change communication sessions, and a control group receiving non-iron cookware. Iron status indicators (hemoglobin, ferritin, serum iron, transferrin saturation, and total iron-binding capacity) will be assessed at baseline, 6 months, and 12 months after the intervention. Patient compliance with iron pot usage will be monitored through food diaries, spot checks, and interviews. Barriers and facilitators of adherence will be explored through qualitative assessments.

**Discussion:**

It is anticipated that women using iron cookware with structured counselling support will demonstrate significant improvements in anemia and iron deficiency indicators compared to controls. This study will also identify the practical, cultural, and economic barriers affecting patient compliance with cooking in iron pots. The findings will contribute to the evidence base for promoting iron cookware as a sustainable intervention in rural population settings. The results will inform community health strategies and align with India’s Anemia Mukt Bharat initiative.

**Trial registration:**

This randomized controlled trial was prospectively registered with the Clinical Trials Registry–India (CTRI) under the registration number CTRI/2025/04/085829, dated April 28, 2025.

**Supplementary Information:**

The online version contains supplementary material available at 10.1186/s13063-025-09202-0.

## Administrative information

**Table:** 

Title {1}	Effect of Consuming Food Cooked in Iron Pots and Behavior Change Communication on Iron Status in Non-Pregnant, Non-Lactating Women: Protocol for Randomized Controlled Trial
Trial registration {2a and 2b}.	This randomized controlled trial was prospectively registered with the Clinical Trials Registry - India (CTRI) under the registration number CTRI/2025/04/085829, dated April 28, 2025.
Protocol version {3}	1.0, dated May 2025
Funding {4}	This research did not receive any specific grants from any funding agency in the public, commercial, or not-for-profit sectors.
Names, affiliations, and roles of protocol contributors {3a}	Umesh G. Kawalkar (Government Medical College & Hospital, Akola, Maharashtra, India) is the Principal Investigator and was responsible for study conception, design, and protocol drafting. Shital Telrandhe (Datta Meghe Institute of Medical Sciences [DMIMS], Wardha) contributed as a co-investigator with responsibility for data collection and community coordination. Mahalaqua Nazli Khatib (DMIMS, Wardha) served as a co-investigator focusing on trial methodology and the behavior change communication component. Shilpa Gaidhane (DMIMS, Wardha) contributed as a co-investigator, providing oversight on nutrition and intervention implementation. Zahiruddin Quazi Syed (DMIMS, Wardha) participated as co-investigator with expertise in epidemiology and biostatistics, and Amar Mankar (DMIMS, Wardha) functioned as co-investigator responsible for field monitoring and logistics. Abhay Gaidhane (DMIMS, Wardha) acted as Senior Mentor and Advisor, providing overall guidance and manuscript review.
Name and contact information for the trial sponsor {3b}	Primary Sponsor: Government Medical College & Hospital, Akola, Maharashtra, India (Department of Community Medicine) Contact Person: Dr. Umesh G. Kawalkar, MD, MPH (Assistant Professor, Principal Investigator) Address: GMC Campus, Ashok Vatika, Akola – 444001, Maharashtra, India Email: umeshkawalkar01@gmail.com Phone: +919730196442 Collaborating Sponsor: Datta Meghe Institute of Medical Sciences (Deemed to be University), Sawangi (Meghe), Wardha, Maharashtra, India Contact Person: Prof. Dr. Abhay M. Gaidhane, MD, PhD (Director – School of Epidemiology & Public Health) Address: DMIMS Campus, Sawangi (Meghe), Wardha – 442001, Maharashtra, India Email: abhaygaidhane@gmail.com Phone: +9765404075
Role of trial sponsor and funders in design, conduct, analysis, and reporting of trial; including any authority over these activities {3c}	This is an investigator-initiated trial. The sponsor (Government Medical College, Akola) and co-affiliated institute (DMIMS Wardha) had no role in the study design, data collection, management, analysis, interpretation, report writing, or decision to publish. The investigators assume full responsibility for the study.
Composition, roles, and responsibilities of the coordinating site, steering committee, endpoint adjudication committee, data management team, and other individuals or groups overseeing the trial, if applicable{5d}	Coordinating Center: Department of Community Medicine, Government Medical College (GMC), Akola. Overall coordination of the trial, field operations, recruitment, follow-up, data entry, and communication between the investigators. Steering Committee: Principal Investigator (Dr. Umesh G. Kawalkar), Co-Investigators (Dr. Shital Telrandhe, Dr. Mahalaqua Nazli Khatib, Dr. Shilpa Gaidhane, Dr. Zahiruddin Quazi Syed, Dr. Amar Mankar), Senior Mentor (Dr. Abhay Gaidhane) provides strategic oversight, ensures adherence to the protocol, monitors progress, reviews interim findings, and addresses operational challenges. Data Management Team: GMC Akola (data entry staff and statistician) and DMIMS Wardha (epidemiology and biostatistics experts) are responsible for data entry, cleaning, validation, and secure storage. Oversees database creation and quality checks and ensures the confidentiality of participants’ data. Endpoint Adjudication Committee: Not applicable (endpoints are objective laboratory measures: hemoglobin, ferritin, iron indices) : N/A Data Monitoring Committee (DMC): Not constituted (minimal risk, non-drug, behavioural/nutritional trial) Safety monitoring incorporated into Steering Committee meetings; ethical oversight through Institutional Ethics Committees (IEC) at GMC Akola and DMIMS Wardha. Ethics Oversight: Institutional Ethics Committee (IEC), Government Medical College Akola; Institutional Ethics Committee, Responsible for initial and continuing ethical approval, monitoring compliance with national/international ethical standards, and reviewing any protocol amendments.
Name of trial registry, identifying number (with URL), and date of registration. If not yet registered, name of intended registry (4)	This randomized controlled trial is prospectively registered with the Clinical Trials Registry - India (CTRI) under registration number CTRI/2025/04/085829, dated 28 April 2025. URL: https://ctri.nic.in/Clinicaltrials/pubview2.php
Where the trial protocol and statistical analysis plan can be accessed (5)	The full trial protocol and Statistical Analysis Plan (SAP) will be made available as supplementary files in the publication of the protocol in *Trials* and deposited in an institutional repository (Government Medical College Akola website) and Shod Ganga (URL:https://shodhganga.inflibnet.ac.in/) with open access.
Where and how the individual de-identified participant data (including data dictionary), statistical code, and any other materials will be accessible (6)	De-identified individual participant data (IPD), data dictionary, and statistical code will be made available in an open data repository (Dryad) within 6 months of primary outcome publication. Requests may also be directed to the PI (e-mail).
Sources of funding and other support (e.g., supply of drugs) (7a)	This study is supported by the Datta Meghe Institute of Higher Education & Research (DMIHER) and Government Medical College Akola, Maharashtra. No commercial entity influenced the study design or reporting.
Financial and other conflicts of interest for principal investigators and steering committee members (7b)	The investigators declare no financial or non-financial competing interests related to this study.
Plans to communicate trial results to participants, healthcare professionals, the public, and other relevant groups (e.g., reporting in trial registry, plain language summary, publication) (8)	The results will be communicated to participants (via community meetings and leaflets in the local language), healthcare professionals (through CME/workshops), the public (press releases, social media), and the scientific community (trial registry updates, peer-reviewed publications, conference presentations). A plain-language summary will be uploaded to the trial registry.

## Introduction

### Background and rationale {9a &9b}

Iron deficiency anemia (IDA) remains a critical public health issue, particularly in developing countries such as India. It disproportionately affects women of reproductive age, children, and other vulnerable populations, leading to impaired cognitive and physical development, reduced productivity, and an increased risk of adverse maternal and neonatal outcomes. Despite the implementation of iron supplementation and fortification programs, the prevalence of anemia remains persistently high, as reported by national surveys such as the National Family Health Survey (NFHS) [1–3]. According to the World Health Organization, globally, approximately 30% of non-pregnant women of reproductive age (15–49 years) suffer from anemia. In India, the prevalence of anemia among non-pregnant, non-lactating women in this age group is approximately 57%, according to the NFHS-5 (2019–21), indicating a severe public health concern [1].

Iron supplementation is widely used to combat anemia; however, its effectiveness as a standalone intervention is often limited by factors such as cost, logistical challenges, poor adherence, and side effects like nausea, vomiting, and constipation. Although fortification of staple foods is cost-effective, it faces similar obstacles, including regulatory constraints and consumer acceptance issues [4]. While biofortification through agricultural innovations holds promise, its impact on key outcomes, such as hemoglobin levels in children, remains uncertain [5]. Dietary diversification is another potential approach; however, access to iron-rich foods, such as meat, and vitamin C-rich produce is often restricted in low-income populations. Additionally, common dietary staples, including cereals, coffee, and tea, contain iron absorption inhibitors, further complicating anemia management [6]. Addressing infections like malaria and intestinal parasites is also crucial, yet these efforts must be integrated within broader nutritional strategies due to the multifaceted nature of anemia [7] Given the persistent burden of IDA, innovative, culturally appropriate, and sustainable interventions are needed. One such strategy is the use of iron cookware, which has gained attention for its low cost, cultural acceptability, and potential to increase dietary iron intake [8]. Cooking in iron pots allows iron to leach into food, potentially enhancing iron consumption, which is an especially relevant intervention in low-resource settings where access to fortified foods or supplements is inconsistent. However, the effectiveness of iron cookware remains inconclusive, as existing studies have reported mixed results. The amount and bioavailability of leached iron depend on several factors, including food acidity, cooking time, and cookware condition [9]. Additionally, cultural practices and user compliance significantly influence the sustained use of these technologies. While some studies have demonstrated modest improvements in hemoglobin levels, others have shown minimal or no impact, highlighting the need for more rigorous, context-specific research [10]

Furthermore, several key variables influencing the efficacy of iron cookware remain unexplored. These include genetic hemoglobinopathies (e.g., thalassemia traits), water quality, and the potential risk of iron overload in certain populations [11]. Most previous studies on iron cookware have focused on children or pregnant women, while non-pregnant, non-lactating women (NPNL) who face a high burden of anaemia remain understudied [8]. In Indian households, this group is usually responsible for daily cooking, making them the most strategic targets for an intervention that uses iron cookware. Importantly, by engaging NPNL women through structured behavior change communication (BCC), this trial not only empowers them but may also indirectly improve iron intake and stores among other family members who consume meals prepared in iron pots [12]. Compliance with the consistent use of iron pots and the practicality of integrating them into daily cooking habits are critical factors that require further investigation. Without addressing these gaps, it is difficult to determine the true public health impact of iron cookware. Therefore, the present study aimed to evaluate the sustained use of iron pots in women of reproductive age, examining their impact on dietary iron intake and biomarkers.

### Objectives {10}

- To evaluate the impact of consuming food cooked in iron pots on iron status indicators, specifically hemoglobin (Hb), serum ferritin, serum iron, transferrin saturation (TSAT), and total iron binding capacity (TIBC) in non-pregnant, non-lactating women of reproductive age group from rural India
- To assess the effect of cooking in iron pots on erythrocyte protoporphyrin, mean corpuscular volume, and C-reactive protein as markers of iron metabolism and inflammation
- To evaluate the compliance rates of Indian households in using iron cookware consistently for meal preparation.
- To identify the practical, cultural, and economic barriers impacting the consistent use of iron cookware in Indian households.

### Patient and public involvement {11}

Patients or members of the public were not involved in the design, conduct, reporting, or dissemination of this study. However, the intervention was informed by existing community dietary practices, and trial results will be shared with participants and local health authorities through community meetings and plain-language summaries.

### Trial design {12}

This is a two-arm, parallel-group, superiority, randomized controlled trial with an allocation ratio of 1:1. Participants will be randomized to receive either iron cookware for daily food preparation plus behaviour change communication package (intervention group) or standard care (control group). Control Group participants will use their usual utensils (such as aluminium or stainless steel) for daily food preparation.

## Methods: participants, interventions and outcomes

### Study setting {13}

This community-based randomized controlled trial (RCT) will be conducted in rural areas of Akola and Wardha districts in Maharashtra, India. One block from each district will be randomly selected for study. The total sample size will be equally distributed across the two sites to ensure geographic and demographic representation. Block selection in both districts will be based on the high prevalence of anemia among women of reproductive age, as reported in the National Family Health Survey (NFHS-5). On average, the prevalence of anemia in Akola and Wardha districts ranged from 54 to 59%. Both districts also represent a diverse rural population with variations in dietary patterns, socioeconomic conditions, and access to healthcare, making them suitable sites for evaluating the impact of iron pot cooking and behaviour change communication (BCC) interventions on iron status. Including two distinct rural settings will enhance the generalizability of the study findings and allow for the assessment of variations in intervention effectiveness across different populations. Randomly selecting blocks within each district will minimize selection bias and ensure a representative sample of non-pregnant, non-lactating women of reproductive age. This approach will generate evidence relevant to broader public health applications, particularly in rural India, where iron deficiency anaemia remains a major concern.

### Eligibility criteria {14a & 14b}

This will include community sensitization and enrolment. The study team will conduct awareness sessions in the community to explain the study’s objectives, potential benefits, and participation requirements. The eligibility of the participants will be assessed based on the following inclusion and exclusion criteria.

### Inclusion criteria

1. Women of reproductive age (18–45 years old).
2. Non-pregnant and non-lactating at the time of enrolment.
3. Residing in the study area for the duration of the intervention.
4. Willingness to cook regularly at home and use the provided iron utensils.
5. No diagnosed anemia treatment (iron supplementation or intravenous iron therapy) in the past 3 months.
6. Participants who agreed to participate and provided informed consent for data collection (biochemical assessments, food diaries, and interviews).

### Exclusion criteria

1. Pregnant or lactating women at the time of enrolment.
2. Women with chronic illnesses (e.g., kidney disease and inflammatory disorders) affectcting iron metabolism.
3. Those with diagnosed or known haematological disorders (e.g., thalassemia, sickle cell anemia) based on haematological investigations such as CBC.
4. Participants who will already using iron cookware regularly before the study.
5. Severe dietary restrictions (e.g., vegan diets or food aversions preventing the consumption of iron-rich foods).
6. Women who are on iron supplements or medications that interfere with iron absorption (e.g., proton pump inhibitors and certain antibiotics).
7. Plans to relocate from the study area during the intervention period.

### Intervention and comparator {15}

- We will adopt a structured approach to ensure both knowledge transfer and practical application, fostering sustained behaviour change for improved iron intake.
- Iron cookware distribution and cooking demonstration: Participants in the intervention group will receive standardized iron cookware for daily use. A set of iron cookware will include the following:
  1. Iron Kadhai (wok): A deep, round-bottomed cooking pan commonly used for stir-frying, sautéing, and deep-frying. This will be the primary vessel for preparing curries, vegetables, and other staple dishes.
  2. Iron Tava (griddle): A flat, circular pan traditionally made for chapatis, rotis, and dosas. Cooking flatbreads on an iron Tava will enhance dietary iron intake due to direct food contact with the surface.
  3. Iron Ganj (pot): A deep cooking pot used for boiling rice, lentils, and other staple foods. Prolonged cooking in iron pots has increased iron leaching into food, improving iron intake.
  4. Iron Sarata (ladle/spoon): A large, sturdy spoon for stirring and serving food. Frequent contact between the ladle and food during cooking may contribute to iron transfer.
- By providing a combination of these commonly used iron utensils, the intervention aims to increase dietary iron intake through routine meal preparation while being culturally and socially appropriate.
- To ensure effective and sustained adoption, they will be trained on:
  - ◦ Optimal cooking techniques—Emphasizing methods that enhance iron release, such as cooking acidic foods (e.g., tomato-based dishes).
  - ◦ Best practices and precautions—Guidance on dos and don’ts, including avoiding excessive scrubbing that may reduce the iron layer and ensuring consistent cookware usage.
  - ◦ Safe handling and maintenance—Instructions on proper care to extend the cookware’s lifespan and ensure safe cooking practices.

### Health education and behaviour change communication (BCC) sessions

The effectiveness of cooking with iron utensils will be evaluated alongside behaviour change communication (BCC) strategies, ensuring that participants adopt and sustain cooking practices that optimize iron absorption. To reinforce knowledge and promote long-term adherence, the study team will conduct group sessions and household visits, focusing on the role of dietary iron, i.e., understanding iron-rich food sources, factors that enhance absorption (e.g., vitamin C), and dietary inhibitors (e.g., tea, coffee, phytates); Anemia Prevention and Management which includes raising awareness about the consequences of iron deficiency and the importance of routine hemoglobin checks and Sustained Behaviour Change for encouraging the habitual use of iron cookware through community-driven approaches, including storytelling and role modelling by early adopters.

Participants in the intervention group will receive fortnightly group BCC sessions for the first 3 months, followed by monthly group sessions for the next 3 months (total, 9 group sessions over 6 months). In addition, monthly individual home visit sessions will be conducted throughout the 6-month intervention period to reinforce key messages, address practical barriers, monitor cookware usage, and offer personalized support. Each group session will last approximately 45–60 min, while home visits will be of 20–30 min duration, ensuring context-specific engagement and continuous motivation.

### Criteria for discontinuing or modifying the intervention {15b}

Participants who become pregnant during the trial will be withdrawn from the primary outcome analysis but may be followed up for safety outcomes with consent. Data collected up to pregnancy will be retained for analysis. Participants may discontinue at their own request or if advised by the study physician for medical reasons.

### Strategies to improve adherence and monitoring {15c}

Adherence will be promoted and monitored through like monthly household visits by field staff to provide reinforcement, troubleshoot issues, and encourage compliance. Food diaries maintained by the participants documented meals prepared using iron cookware. Spot checks during unannounced household visits to observe real-time usage. Structured surveys and interviews to explore barriers and facilitators. Compliance will be defined as use of the provided iron cookware for at least one main meal/day on ≥ 4 days per week, maintained for ≥ 70% of the intervention period. This threshold is based on prior iron-cookware RCTs. Focus group discussions (FGDs) to further refine adherence strategies and address community concerns. Adherence outcomes will be reported, and both per-protocol and intention-to-treat analyses will be performed.

### Concomitant care permitted/prohibited {15d}

Participants will be asked to avoid initiating iron supplementation or other medications that affect iron metabolism during the trial period, unless medically indicated. Any such use will be recorded, and participants will remain in ITT analyses.

### Outcomes {16}

The outcome measures and assessment time points are summarized in Table 1. Blood samples will be collected at each time point for the biochemical analysis of iron status markers. Dietary intake and adherence to cookware use will be monitored using structured questionnaires and periodic household visits.

**Table 1:** Summary of primary, secondary, and exploratory outcomes with measurement variables, analysis metrics, aggregation methods, and time points (baseline to 12 months)

Outcome type	Measurement variable	Analysis metric	Method of aggregation	Time points
Primary outcome	Serum ferritin (µg/L), CRP-adjusted	Mean change from baseline	Mean ± SD, mean difference (95% CI)	Baseline, 6 months, 12 months
Secondary outcomes	Haemoglobin (g/dL)	Mean change from baseline	Mean difference (95% CI)	Baseline, 6 months, 12 months
	Soluble transferrin receptor (mg/L)	Mean change from baseline	Mean difference (95% CI)	Baseline, 6 months, 12 months
	Transferrin saturation (%)	Mean change from baseline	Mean difference (95% CI)	Baseline, 6 months, 12 months
	Anaemia prevalence (Hb < 12 g/dL, WHO)	Proportion with anaemia	Risk difference, risk ratio (95% CI)	Baseline, 6 months, 12 months
	Nutrition knowledge and practice score	Change in score	Mean difference	Baseline, 6 months, 12 months
	Adherence to iron cookware use (≥ 1 main meal/day, ≥ 4 days/week)	Proportion adherent	Percentage adherence	Monthly monitoring; summarised at 6 months, 12 months
	Adverse events (GI symptoms, iron overload, other harms)	Proportion with ≥ 1 adverse event	Frequency counts, percentages	Ongoing; summarised at 3, 6, and 12 months
Exploratory outcomes	Cost-effectiveness (per unit Hb and ferritin increase)	Incremental cost-effectiveness ratio (ICER)	ICER	6 months, 12 months
	Dietary modifiers (vitamin C intake, tea/coffee, phytates)	Effect modification	Subgroup analyses	Baseline, 6 months, 12 months
	Subgroup analysis by baseline anaemia	Effect difference	Interaction tests	6 months, 12 months
	Community-level adoption of cookware post-trial	Proportion of households continuing usage	Adoption rate (%)	Endline at 12 months; post-trial sustainability follow-up

### Harms {17}

Harms included any adverse event (AE) temporally associated with trial participation/intervention, whether or not considered related, and serious adverse events (SAEs) (death, life-threatening event, inpatient hospitalization/prolongation, significant disability, or important medical events The expected AEs for this trial include minor gastrointestinal symptoms (e.g., nausea, constipation, abdominal discomfort, and dark stools); skin/contact events (e.g., minor burns while cooking); and laboratory abnormalities consistent with changes in iron status. Potential though unlikely safety signals of excess iron will be monitored using ferritin and transferrin saturation (TSAT). The participant timeline, including schedule of enrollment, interventions, and assessments, is shown in Table 2.

**Table 2:**
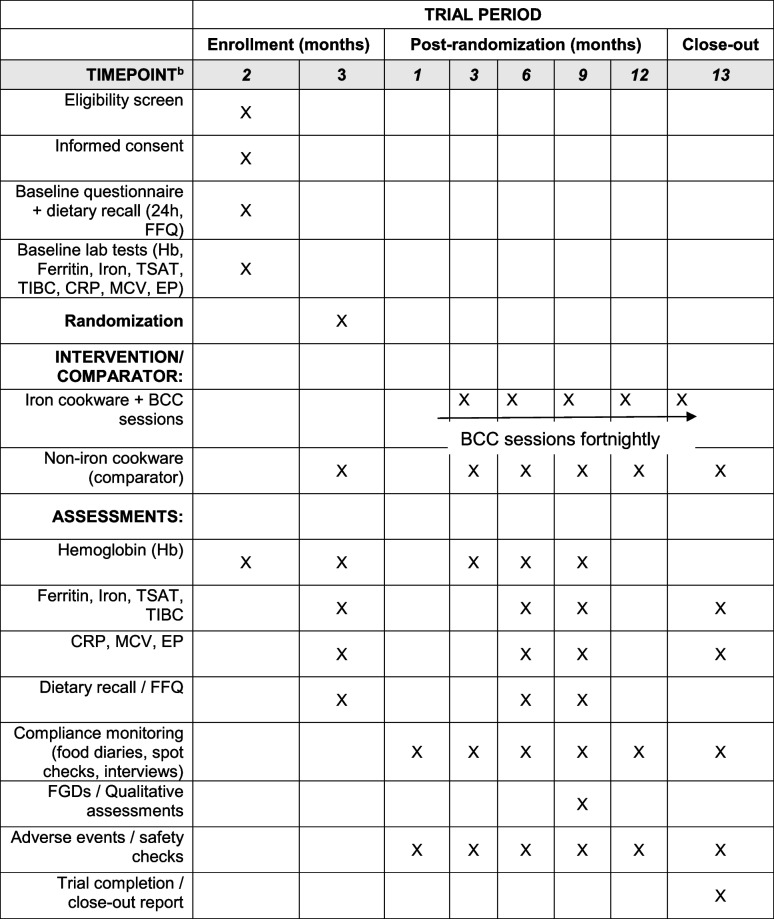
Participant timeline: schedule of enrollment, interventions, and assessments [17]^a^

^a^ Recommended content can be displayed using various schematic formats

^b^ List target timepoints and acceptable time windows in this row (e.g., 30 ± 3 days)

^c^ Arrow indicates continuous delivery of intervention (e.g., Use of iron pots & BCC)

#### Ascertainment and frequency

Harms will be assessed systematically at every study contact (baseline, 3, 6, and 12 months) and during monthly home visits in the intervention period, using a structured checklist and open-ended queries. Participants may also self-report harms at any time via a study phone number/WhatsApp. Field workers will document onset, duration, severity, actions taken, and outcome; supervisors verify within 48 h.

#### Severity grading and relatedness

Severity will be graded using Common Terminology Criteria for Adverse Events, Version 5.0 (CTCAE v5.0) (Grades 1–5) adapted for community settings [13]. Causality will be assigned by the study physician using WHO-UMC categories (certain, probable/likely, possible, unlikely, conditional/unclassified, unassessable) [14].

#### Clinical thresholds and actions

If ferritin rises above a pre-specified threshold (e.g., > 150 µg/L in women) and TSAT > 45%, the participant will be reviewed by the study physician; cookware use may be paused and repeat labs obtained. Any burn beyond first-degree or requiring medical care will be recorded as ≥ Grade 2 and treated/referral arranged. Initiation of therapeutic iron by outside providers will be recorded (concomitant care) and monitored for GI AEs [15, 16].

### Reporting and timelines

- Serious adverse events (SAEs): notified to the Principal Investigator and ethics committee within 24 h; reported to the sponsor and registry per local regulations.
- Adverse events (AEs): summarized and reported monthly to the internal safety monitor and quarterly to the ethics committee.
- Unanticipated problems posing risk to participants or community will be reported promptly and corrective actions documented.

#### Data safety oversight

An independent safety monitor/DSMB (if required by IEC) will review blinded AE/SAE listings after ~ 25% and ~ 50% of expected person-time. The DSMB/sponsor may recommend protocol modifications, temporary suspensions, or terminations for safety concerns.

#### Medical management and compensation

Participants with AEs receive appropriate first aid, referral, and follow-up at no cost as per institutional policy and national regulations. Compensation for research-related injury will follow institutional ethics guidelines.

#### Documentation

All harms will be recorded in source notes and the eCRF, with MedDRA coding (if applicable), and included in interim/final reports and trial publications.

### Participant timeline {18}

#### Sample size {19}

The sample size was calculated based on hemoglobin (Hb) as the key outcome variable. Considering a standard deviation (SD) of 1.2 g/dL and a minimum detectable difference (Δ) of 0.5 g/dL between the intervention and control groups, the required sample size was estimated to provide 80% power (*β* = 0.20) at a two-sided significance level of 5% (*α* = 0.05, 95% confidence level). The sample size was adjusted accordingly to account for an anticipated 10% attrition due to loss to follow-up, pregnancy, or withdrawal. The final required sample size is 202 participants, with 101 participants allocated to the intervention group and 101 participants to the control group [18, 19]. Since we will pool the data from two sites for the main outcome measures, the total sample size will be equally divided into two study sites.

The sampling strategy for this study involves a multi-stage approach to ensure methodological rigor and reduce potential biases. Initially, eligible villages will be purposefully selected from study areas based on predefined inclusion criteria such as population size, accessibility, and anemia prevalence data, if available. Within each selected village, a household listing will be conducted to identify households with at least one eligible non-pregnant, non-lactating (NPNL) woman of reproductive age. In instances where more than one eligible woman resides in a single household, one participant will be selected using simple random selection methods—such as a coin toss or random number generation—to prevent intra-household clustering and to maintain the independence of observations. This ensures that each household contributes only one participant to the study. Following this, the identified households will be subjected to block randomization, as previously described, to assign participants into intervention and control groups in balanced proportions. This sampling framework enables equitable group allocation, reduces selection bias, and ensures that the study sample is representative of the target population within the selected geographic context.

### Recruitment {20}

Recruitment will be done through village-level sensitization. Eligible households will be listed, and one woman randomly selected per household. Block randomization will ensure group balance.

### Randomization and allocation

#### Sequence generation {21a}

Participants will be randomly assigned to either the intervention or control group using a block randomization approach to ensure balanced allocation. Randomization will be conducted in small, variable block sizes (e.g., blocks of 4), with possible sequences such as ABAB, BABA, AABB, or BBAA (where A = intervention, B = control). A pre-generated randomization list will be prepared by an independent statistician to guarantee equal group sizes and minimize allocation bias.

### Type of randomization {21b}

Restricted randomization (block randomization) with a 1:1 allocation ratio will be used. The block size and sequence order will remain concealed from the field team to prevent predictability.

### Allocation concealment mechanism {22}

Allocation will be concealed using sequentially numbered, opaque, sealed envelopes (SNOSE), managed exclusively by the independent statistician. Each envelope will contain the allocation code and will be opened only after eligibility confirmation and participant consent.

### Implementation {23}

Field investigators will be responsible for participant enrolment. Randomization and envelope management will be handled by an independent statistician, while intervention allocation will be assigned by a trial coordinator. This separation of responsibilities will ensure that the investigators enrolling participants have no prior knowledge of the sequence.

### Blinding {24}

#### Who will be blinded {24a}

Due to the visible nature of the cookware intervention, participants and field investigators responsible for delivering the intervention cannot be blinded. However, blinding will be maintained for specific trial personnel. Outcome assessors, including laboratory technicians analyzing blood samples for hemoglobin, ferritin, soluble transferrin receptor (sTfR), transferrin saturation, and C-reactive protein (CRP), will remain blinded to group allocation. In addition, data analysts/statisticians will conduct analyses using coded datasets in which group identifiers have been removed, thereby preserving allocation concealment during data analysis.

### How blinding will be achieved {24b}

To maintain blinding, all blood samples will be labelled with anonymized study codes rather than allocation labels. Therefore, the laboratory staff will be unaware of the participants’ group assignments. Similarly, the trial statistician will receive only coded datasets where groups are labelled “Group A” and “Group B.” The cookware used in the intervention arm differed visibly from the control cookware (iron vs. non-iron); hence, participant blinding was not feasible.

### Circumstances for unblinding {24c}

Unblinding of laboratory staff or statisticians is not anticipated. However, if a participant experiences a serious adverse event suspected to be related to the intervention (e.g., severe gastrointestinal symptoms, potential iron overload), the Principal Investigator (PI) may authorize unblinding to confirm group allocation for appropriate clinical management. The procedure will involve the PI contacting the independent statistician who generated the randomization sequence; group assignment will be disclosed only to the treating physician and ethics committee, and not to outcome assessors or data analysts.

### Data collection methods {25}

#### Plans for assessment and collection of outcomes {25a}

Data collection will include both quantitative and qualitative measures to evaluate iron status, dietary intake, adherence, and perceptions of the intervention. Quantitative data will be obtained through structured questionnaires to capture sociodemographic characteristics, health history, and dietary practices, as well as a 24-h dietary recall and Food Frequency Questionnaire (FFQ) to estimate dietary iron intake and food patterns. Laboratory investigations will be carried out at an NABL-accredited laboratory, where trained phlebotomists will collect blood samples for analysis of hemoglobin (Hb), serum ferritin, serum iron, soluble transferrin receptor (sTfR), transferrin saturation (TSAT), total iron binding capacity (TIBC), C-reactive protein (CRP), erythrocyte protoporphyrin, and mean corpuscular volume (MCV). These parameters will be measured at baseline, 6 months, and 12 months, with Hb and ferritin additionally assessed at 3 months. Duplicate testing will be performed on 10% of samples to ensure validity. Qualitative data will be collected through semi-structured interviews and focus group discussions (FGDs) to explore perceptions, facilitators, and barriers to cookware adoption and BCC acceptability, supplemented by field observations recorded during household visits to triangulate adherence and contextual factors. All assessors and laboratory staff will be trained and blinded to group allocation, and standard operating procedures (SOPs) will be followed at all stages to ensure consistency and data quality.

### Plans to promote participant retention and follow-up {25b}

Retention will be promoted through regular household visits, community meetings, and reminder phone calls/WhatsApp messages before scheduled assessments. Monthly home visits will reinforce adherence, troubleshoot problems, and monitor compliance. Compliance will be assessed by Food diaries maintained by participants; Unannounced spot checks by field staff, and Monthly supervisory visits. Compliance is defined as use of the provided iron cookware for ≥ 1 main meal per day on ≥ 4 days per week, sustained for ≥ 70% of the intervention period. Participants not meeting this criterion will be considered non-compliant for per-protocol analysis but will remain in the intention-to-treat (ITT) analysis. Outcome data (Hb, ferritin, and other biomarkers) will be collected for all randomized participants, even if they discontinue or deviate from the intervention, unless consent is withdrawn entirely.

### Data management {26}

Data will be collected using paper-based case report forms (CRFs) and subsequently entered into a secure electronic database. To ensure accuracy and reliability, double data entry and validation will be performed, and range and consistency checks (e.g., hemoglobin values < 5 g/dL or > 18 g/dL) will be applied to identify implausible entries. Each participant will be assigned a unique coded identifier, with the linkage file maintained separately by the Principal Investigator in a password-protected system. Paper CRFs will be stored in locked cabinets accessible only to authorized staff, while electronic datasets will be encrypted and restricted to study personnel with appropriate clearance. Weekly data backups will be maintained on institutional servers. All personal identifiers will be removed prior to analysis, and no identifiable information will be disclosed in publications. A detailed data management standard operating procedure (SOP) will be archived and made available upon request.

### Statistical methods {27}

#### Primary and secondary outcomes {27a}

Descriptive statistics will be used to summarize baseline characteristics. Continuous variables will be expressed as mean (SD) or median (IQR) as appropriate, and categorical variables as frequencies and percentages. Baseline differences between intervention and control groups will be assessed using independent *t*-tests (or Mann–Whitney *U* tests for non-normal data) and chi-square or Fisher’s exact tests for categorical variables. Standardized mean differences (SMDs) will be calculated, with an SMD < 0.1 indicating adequate baseline balance.

Continuous outcomes such as haemoglobin (Hb), serum ferritin, soluble transferrin receptor (sTfR), and transferrin saturation (TSAT) will be analyzed using linear mixed-effects models with fixed effects for group, time, and group × time interaction, and random effects for participants (and clusters if applicable). Binary outcomes such as anaemia prevalence, adherence, and adverse events will be analyzed using mixed-effects logistic regression. Risk ratios, odds ratios, mean differences, and 95% confidence intervals will be reported. The significance level will be set at *p* < 0.05 (two-tailed). Analyses will be performed using R software.

To provide a comprehensive evaluation of iron status, additional biomarkers will be assessed, including total iron-binding capacity (TIBC), erythrocyte protoporphyrin, mean corpuscular volume (MCV), and C-reactive protein (CRP). These indicators will enable differentiation between iron deficiency, anaemia of inflammation, and other causes of low Hb. Effect size (Cohen’s d) will be calculated for the primary outcomes (Hb and ferritin) to quantify the magnitude of intervention impact**.**

### Analysis population {27b}

All randomized participants will be included in the analysis according to the group to which they were allocated (intention-to-treat, ITT). A per-protocol analysis will also be conducted for adherence-related outcomes, where compliance is defined as use of the provided iron cookware for ≥ 1 main meal per day on ≥ 4 days per week for at least 70% of the intervention period.

### Missing data {27c}

Missing outcome data will be addressed using maximum likelihood estimation under the missing-at-random assumption. Multiple imputation with 20 datasets will be performed as a sensitivity analysis. Reasons for missingness (e.g., pregnancy, migration, withdrawal) will be documented. Additional sensitivity analyses will include complete-case analysis and per-protocol analysis to evaluate robustness of findings.

### Additional analyses {27d}

Pre-specified subgroup analyses will assess effect modification by baseline anaemia status (anaemic vs non-anaemic), dietary iron intake categories, and CRP-adjusted vs unadjusted ferritin. Mixed-effects regression models will be applied with adjustment for covariates including age, body mass index (BMI), baseline iron status, dietary intake, inflammation (CRP), and socioeconomic status. Sensitivity analyses will test the robustness of results to exclusion of participants with elevated CRP, alternative adherence thresholds, and different imputation strategies. An exploratory cost-effectiveness analysis will be conducted, estimating the incremental cost per unit increase in haemoglobin (g/dL) and ferritin (µg/L).

### Data monitoring committee {28}

#### Composition and role {28a}

A formal independent Data Monitoring Committee (DMC) will not be established because the intervention (iron cookware and behaviour change communication) is non-pharmacological, community-based, and considered low-risk. Oversight will instead be provided by the Institutional Ethics Committee (IEC), Government Medical College Akola, Maharashtra, which functions independently from the sponsor and funder and has no conflicts of interest related to the trial. The IEC will review periodic safety and progress reports.

### Interim analyses and stopping guidelines {28b}

No interim efficacy analyses are planned given the modest sample size and limited duration of the trial. However, interim safety monitoring will be undertaken. Serious adverse events (SAEs) and any unexpected safety signals (e.g., severe gastrointestinal events, evidence of iron overload) will be reported immediately to the Principal Investigator and IEC. The IEC retains the authority to recommend protocol modification, temporary suspension, or trial termination in case of safety concerns.

### Trial monitoring {29}

Trial monitoring will be conducted by the study’s internal monitoring team, which will include the Principal Investigator, Co-investigators, and field supervisors. The team will undertake weekly reviews of field logs and enrolment registers, along with monthly household spot checks to verify adherence and data quality. Progress, completeness of data, and safety reporting will be assessed in quarterly project review meetings, while annual reports summarising trial conduct, safety updates, and protocol compliance will be submitted to the Institutional Ethics Committee (IEC). Given the pragmatic and low-risk nature of the intervention, no external contract research organization (CRO) monitoring is planned.

### Research ethics approval {30}

The study protocol has been reviewed and approved by the Institutional Ethics Committee (IEC), Government Medical College Akola, Maharashtra, India. The protocol was reviewed under the Department of Pharmacology and received approval in the IEC meeting held on 25 November 2025. The decision was conveyed on the same date, and the study was assigned IEC Serial No. 123/2025. The approval is valid for the duration of the research, from 01 June 2024 to 31 May 2027. The trial will be conducted in accordance with the principles of the Declaration of Helsinki, Good Clinical Practice (GCP), and applicable national regulations.

### Protocol amendments {31}

Any important modifications to the protocol (e.g., changes to eligibility criteria, outcomes, sample size, or analysis plan) will be submitted for prior approval to the IEC. Approved amendments will also be updated in the trial registry (CTRI/ClinicalTrials.gov) and communicated to the sponsor, investigators, and study team. If required, participants will be re-consented before implementation of changes that directly affect their participation.

### Consent or assent {32}

#### Informed consent/assent process {32a}

Written informed consent will be obtained from all eligible participants prior to enrolment. Trained field investigators, under the supervision of the study investigators, will explain the trial purpose, procedures, potential risks, and benefits in the local language (Marathi/Hindi). Participants will have the opportunity to ask questions before signing or thumb-impressing the consent form.

### Additional consent provisions {32b}

Consent forms will include a section on the collection, storage, and use of biological specimens (blood samples) strictly for trial-related analyses (haemoglobin, ferritin, transferrin indices, CRP). No additional ancillary genetic or biobanking studies are planned. If future use is envisaged, separate consent will be obtained.

### Confidentiality {33}

**Fig. 1:**
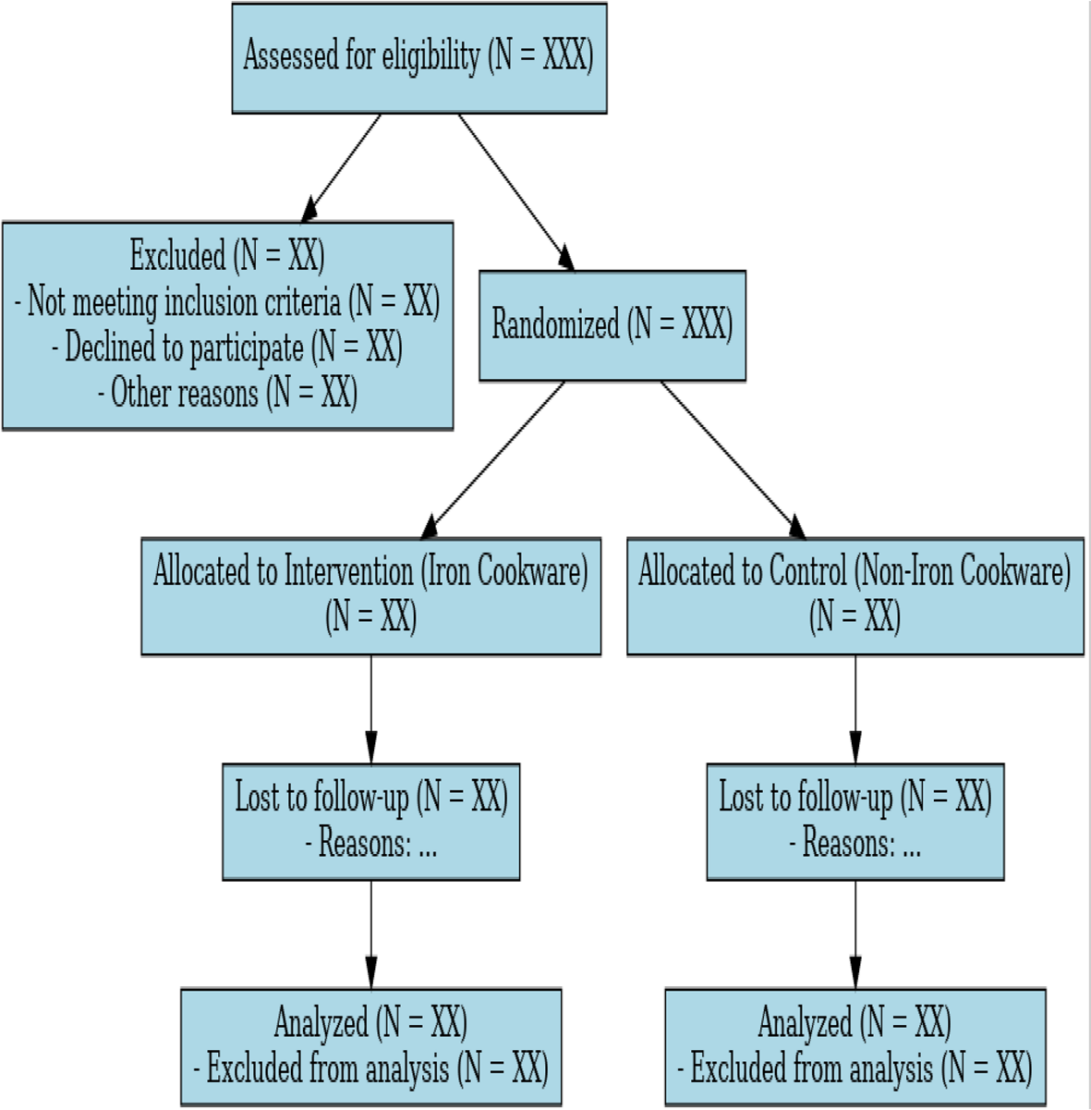
CONSORT flow diagram showing the enrolment, randomization, allocation, follow-up, and analysis of participants in the trial

Personal identifiers (name, address, phone number) will be collected solely for participant follow-up and will be stored separately from study data. Each participant will be assigned a unique study identification code, and only coded data will be used for analysis and dissemination. Paper case report forms (CRFs) will be stored in locked cabinets accessible only to authorized staff, and electronic datasets will be password-protected, encrypted, and stored on secure institutional servers. Data will be retained for at least 5 years after trial completion, after which it will be destroyed in line with institutional policies. The participant flow through the trial is illustrated in Fig. 1 (CONSORT flow diagram).

### Ancillary and post-trial care {34}

At the end of the study, participants will be provided with their iron status reports. Women identified with anaemia will be referred to local primary health centres for free treatment as per the Government of India’s Anaemia Mukt Bharat (AMB) programme. In the event of any research-related harm, appropriate medical management will be provided free of cost, and compensation will be offered as per institutional ethics committee guidelines and national regulations.

## Discussion

The study will assess improvements in iron status indicators haemoglobin, serum ferritin, transferrin saturation, and total iron-binding capacity among women consuming food cooked in iron pots. It will evaluate compliance and identify cultural, economic, and practical barriers to sustained use. Beyond clinical outcomes, the study aims to establish iron cookware as a cost-effective, scalable strategy to combat iron deficiency anemia in India. Findings will inform public health policies through scientific evidence, policy briefs, and community education tools, supporting its integration into “Anaemia Mukta Bharat” programs. Key deliverables include publications, policy recommendations, and training materials for community health workers [12, 19].

Our study has a number of important strengths. By testing the use of iron cookware alongside behaviour change communication, we explore a simple, low-cost and culturally acceptable approach that could be integrated into everyday cooking practices in rural households. Unlike many previous programs that focus mainly on pregnant or lactating women, we deliberately chose to include non-pregnant, non-lactating women, who also carry a high burden of anaemia but are often neglected in public health interventions [20]. The trial follows a rigorous randomized design, with clear adherence to the SPIRIT 2025 guidelines, and collects a wide range of outcomes from blood markers such as haemoglobin and ferritin to detailed dietary information and qualitative insights on compliance [21]. Because the intervention is delivered through community and primary health settings, its results may be directly useful for policy and program scale-up [3]. At the same time, there are limitations. As cookware use cannot be blinded, participants know which group they belong to, and this could influence their behaviour [19]. Dietary recalls and self-reported compliance may not always be accurate, despite efforts to validate them. Finally, the trial is confined to rural blocks of Maharashtra and follows women for only 12 months, so its findings may not fully apply to other settings or reflect longer-term sustainability [22].

This study anticipates generating new knowledge on the effectiveness of iron cookware in improving iron status across diverse demographic groups in Indian households. By evaluating changes in hemoglobin and iron biomarkers, it will provide evidence on the bioavailability of iron leached during cooking [9]. The research will also identify compliance rates, user acceptability, and practical barriers, offering insights into cultural and behavioural factors influencing cookware adoption. Additionally, the findings will contribute to public health strategies by comparing the intervention with existing anemia prevention measures, ultimately providing actionable recommendations for addressing iron deficiency anemia in resource-limited settings [8, 9].

### Trial status

- Protocol version: 1.0, dated May 2025
- Recruitment starts: December 2025
- Expected completion: December 2027

## Supplementary Information

Additional file 1: SPIRIT 2025.

## Data Availability

The datasets generated and analyzed during the current study are not publicly available due to participant confidentiality and privacy protections but are available from the corresponding author upon reasonable request.

## Abbreviations

IDA: Iron deficiency anemia
RCT: Randomized controlled trial
BCC: Behavior change communication
Hb: Hemoglobin
TSAT: Transferrin saturation
TIBC: Total iron binding capacity
MCV: Mean corpuscular volume
CRP: C-reactive protein
NPNL: Non-pregnant, non-lactating
NFHS: National Family Health Survey
IEC: Institutional Ethics Committee
FGD: Focus group discussion

## References

[1] Sappani M, Mani T, Asirvatham ES, Joy M, Babu M, Jeyaseelan L. Trends in prevalence and determinants of severe and moderate anaemia among women of reproductive age during the last 15 years in India. PLoS ONE. 2023;18(6):e0286464. 10.1371/journal.pone.0286464. 37262022 10.1371/journal.pone.0286464 PMC10234534

[2] Let S, Tiwari S, Singh A, Chakrabarty M. Prevalence and determinants of anaemia among women of reproductive age in Aspirational Districts of India: an analysis of NFHS 4 and NFHS 5 data. BMC Public Health. 2024;24:17789. 10.1186/s12889-024-17789-3. 10.1186/s12889-024-17789-3 PMC10860231 38347505

[3] GM Brittenham, G Moir-Meyer, KM Abuga, A Datta-Mitra, C Cerami, R Green, et al. Biology of anemia: a public health perspective. J Nutr. 2023;153(Suppl 1 S7):28. 10.1016/j.jnut.2023.07.005. 10.1016/j.tjnut.2023.07.018 PMC7618251 37778889

[4] Sharma S, Khandelwal R, Yadav K, Ramaswamy G, Vohra K. Effect of cooking food in iron-containing cookware on increase in blood hemoglobin level and iron content of the food: a systematic review. Nepal J Epidemiol. 2021;11(2):994–1007. 10.3126/nje.v11i2.38487. 34290890 10.3126/nje.v11i2.36682 PMC8266402

[5] Hurrell R, Egli I. Iron bioavailability and dietary reference values. Am J Clin Nutr. 2010;91(5):1461S-1467S. 10.3945/ajcn.2010.28674D. 20200263 10.3945/ajcn.2010.28674F

[6] Zimmermann MB, Hurrell RF. Nutritional iron deficiency. Lancet. 2007;370(9586):511–20. 10.1016/S0140-6736(07)61235-5. 17693180 10.1016/S0140-6736(07)61235-5

[7] Gera T, Sachdev HS, Boy E. Effect of iron-fortified foods on hematologic and biological outcomes: systematic review of randomized controlled trials. Am J Clin Nutr. 2012;96(2):309–24. 10.3945/ajcn.111.033191. 22760566 10.3945/ajcn.111.031500

[8] Geerligs PD, Brabin BJ, Omari AAA. Food prepared in iron cooking pots as an intervention for reducing iron deficiency anaemia in developing countries: a systematic review. J Hum Nutr Diet. 2003;16(4):275–81. 10.1046/j.1365-277X.2003.00447.x. 12859709 10.1046/j.1365-277x.2003.00447.x

[9] Shi C, Zhe G, Ding X, Meng Q, Li J, Deng L. Effect of cooking conditions on iron release from pots and development of kinetic models for iron supplementation in NIPs. Curr Res Food Sci. 2024;9:100830. 10.1016/j.crfs.2023.100830. 39286428 10.1016/j.crfs.2024.100830 PMC11403410

[10] Alves C, Saleh A, Alaofè H. Iron-containing cookware for the reduction of iron deficiency anemia among children and females of reproductive age in low- and middle-income countries: a systematic review. PLoS One. 2019;14(9):e0221094. 10.1371/journal.pone.0221094. 31479458 10.1371/journal.pone.0221094 PMC6719866

[11] Heath ALM, Skeaff CM, O’Brien SM, Gibson RS, Williams SM. Can dietary treatment of non-anemic iron deficiency improve iron status? J Am Coll Nutr. 2001;20(5):477–84. 10.1080/07315724.2001.10719056. 11601562 10.1080/07315724.2001.10719056

[12] National Cancer Institute. CTEP trial development and conduct. 2025. Available from: https://dctd.cancer.gov/research/ctep-trials/trial-development. Cited 2025 Oct 3.

[13] Shukla AK, Jhaj R, Misra S, Ahmed SN, Nanda M, Chaudhary D. Agreement between WHO-UMC causality scale and the Naranjo algorithm for causality assessment of adverse drug reactions. J Family Med Prim Care. 2021;10(9):3303–8. 10.4103/jfmpc.jfmpc_1534_20. 34760748 10.4103/jfmpc.jfmpc_831_21 PMC8565125

[14] Ogilvie C, Fitzsimons K, Fitzsimons EJ. Serum ferritin values in primary care: are high values overlooked? J Clin Pathol. 2010;63(12):1124–1126. 10.1136/jcp.2010.079541. 10.1136/jcp.2010.083188 20947869

[15] Li X, Duan X, Tan D, Zhang B, Xu A, Qiu N, et al. Iron deficiency and overload in men and women of reproductive age, and pregnant women. Reprod Toxicol. 2023;118:108381. 10.1016/j.reprotox.2023.108381. 37023911 10.1016/j.reprotox.2023.108381

[16] Chan AW, Boutron I, Hopewell S, Moher D, Schulz KF, Collins GS, et al. SPIRIT 2025 statement: updated guideline for protocols of randomised trials. Lancet. 2025. 10.1016/S0140-6736(25)00770-6. 10.1016/S0140-6736(25)00770-6 42290521

[17] Julious SA. Sample sizes for clinical trials with normal data. Stat Med. 2004;23(12):1921–1986. 10.1002/sim.1783. 10.1002/sim.1783 15195324

[18] Rai RK, Fawzi WW, Barik A, Chowdhury A. The burden of iron-deficiency anaemia among women in India: how have iron and folic acid interventions fared? WHO South East Asia J Public Health. 2018;7(1):18–23. 10.4103/2224-3151.239419. 10.4103/2224-3151.228423 29582845

[19] Palupi L, Schultink W, Achadi E, Gross R. Effective community intervention to improve hemoglobin status in preschoolers receiving once-weekly iron supplementation. Am J Clin Nutr. 1997;65(4):1057–1061. 10.1093/ajcn/65.4.1057. 10.1093/ajcn/65.4.1057 9094893

[20] Kumar SB, Arnipalli SR, Mehta P, Carrau S, Ziouzenkova O. Iron deficiency anemia: efficacy and limitations of nutritional and comprehensive mitigation strategies. Nutrients. 2022;14(14):2976. 10.3390/nu14142976. 10.3390/nu14142976 PMC9315959 35889932

[21] Zlotkin SH, Christofides AL, Hyder SMZ, Schauer CS, Tondeur MC, Sharieff W. Controlling iron deficiency anemia through the use of home-fortified complementary foods. Indian J Pediatr. 2004;71(11):1015–1019. 10.1007/BF02828118. 10.1007/BF02828118 15572823

[22] Heath ALM, Skeaff CM, O’Brien SM, Gibson RS, Williams SM. Can dietary treatment of non-anemic iron deficiency improve iron status? J Am Coll Nutr. 2001;20(5):477–484. 10.1080/07315724.2001. 10.1080/07315724.2001.10719056 11601562

